# The Experience of Chinese Couples Undergoing In Vitro Fertilization Treatment: Perception of the Treatment Process and Partner Support

**DOI:** 10.1371/journal.pone.0139691

**Published:** 2015-10-02

**Authors:** Li-Ying Ying, Lai Har Wu, Alice Yuen Loke

**Affiliations:** 1 School of Nursing, The Hong Kong Polytechnic University, Hong Kong, China; 2 School of Nursing, Zhejiang Chinese Medical University, Hangzhou, Zhejiang, China; China Agricultural University, CHINA

## Abstract

**Background:**

Couples undergoing In Vitro Fertilization (IVF) Treatment suffer as dyads from the stressful experience of the painful treatment and the fear that the IVF cycle will fail. They are likely to report that their marital relationship has become unstable due to the prolonged period of treatment.

**Methods:**

This is a qualitative study that was conducted to explore the experiences that Chinese couples have had with IVF treatment, especially their perceptions of the process and the support between couples.

**Results:**

The interviews revealed that couples suffered from the process, experiencing physical and emotional pain, struggling with the urgency and inflexibility of bearing a child, and experiencing disturbances in their daily routines and work. The participants described how they endured the hardships as a couple and how it affected their relationship. The couples felt that sharing feelings and supporting each other contribute to psychological well-being and improves the marital relationship. They also identified some unfavorable aspects in their partner relationship. They were ambivalent about receiving social support from friends and family members.

**Conclusions:**

With the couples indicating that the support that they received from each other affected their experience during the treatment process, it is suggested that a supportive intervention that focuses on enhancing the partnership of the couples and dealing with their inflexibility on the issue of bearing a child might result in improvements in the psychological status and marital relationship of infertile couples undergoing IVF treatment.

## Introduction

It is estimated that, worldwide, the primary infertility rate of women aged 20–44 years is 1.9%. The prevalence of infertility varies in different countries. In China, about1.3% of women of reproductive age are affected [1]. To fulfill the desire for parenthood, 50% of infertile couples would seek medical treatment, which could initially include medication and/or surgery [2]. If these first-line treatments do not work or are deemed inappropriate, about 3% of these couples will be recommended to undergo assisted reproductive technologies (ARTs). In Vitro Fertilization (IVF) comprises more than 99% of ARTs, with a success rate of 16.6–20.2% [3].

When treatment begins, the couples have to endure a variety of treatments, including ovarian stimulation, regular monitoring, oocyte retrieval, embryo transfer, and progesterone supplementation [4]. The treatment appointments, investigations, and injections can greatly disturb a couple’s daily routines [5]. It can therefore be expected that couples, as a unit, suffer from the stressful experience of infertility, as they endure the painful treatment and the fear that that it will fail.

It has also been revealed that couples seeking IVF treatment are more likely than non IVF couples to report that their relationship has become unstable, due to the prolonged nature and the demands of the treatment [6, 7]. It has been shown that the marital and sexual satisfaction of infertile couples can deteriorate because of infertility treatments, and that couples can manifest marital maladjustment even three years after the treatments have ended [6]. Another study revealed that both men and women reported a lower level of satisfaction with their marital relationship after three cycles of infertility treatments that did not lead to a successful pregnancy [8]. Couples expressed difficulty in handling their sexual life, which had been compromised to meet the schedule required because of IVF treatment[9]. The toll on the couples’ relationship as a result of infertility has been reported as the major source of stress leading to the termination of IVF treatment [10]. On the other hand, the marital relationship can also be a protective factor for couples enduring the different stages of the IVF cycle [11], especially for women with unsuccessful IVF outcomes [12].

The relationship of the partners and the support that they give each other will affect their experience during the treatment. Studies have explored the experiences of women/couples relating to IVF treatment. These have mainly focused on the impact of social context on infertility [9], specifically on the two weeks spent waiting for the results on the pregnancy following the embryo transfer[13], the support from other IVF patients via the Internet [14], and life after unsuccessful or terminated treatment [15–17]. However, the experience of such couples with regard to mutual support of partners and their unmet support needs have not been explored or well understood.

This is a qualitative study intended to explore Chinese couples’ experience of IVF treatment, especially their perceptions of the treatment process and the support between marital partners. The results of this study will shed light on the needs of these couples, so that a supportive program can be developed.

## Methods

### Design and setting

The study adopted a qualitative descriptive approach to obtain a better understanding of the experiences of couples undergoing IVF treatment. This approach is appropriate when straight descriptions of a phenomenon are desired [18]. Data collection was conducted through in-depth interviews of individual couple-dyads because of the sensitive nature of infertility. The male and female partner of a couple was interviewed together to gain a clear picture of their interaction, shared experiences, and support for each other.

### Sampling and data collection

The study targeted infertile couples who have undergone at least one cycle of IVF treatment in the past twelve months. Convenience sampling was used in recruiting participants, and until data saturation was reached.

The participants were recruited from a reproductive medical center at a university hospital in the city of Hangzhou, the capital and largest city of the Zhejiang province, at the east coastal of China. Participants were referred through nurses of the clinic. Interviews were conducted in a quiet room of the outpatient clinic whenever it was convenient for the informants. There was no one else present during the interview. Each interview lasted for 60 to 90 minutes, and was conducted by first author, a female PhD student, with experience in qualitative interviewing. All of the interviews were conducted in Chinese, audio-taped, and transcribed verbatim within two weeks after the interviews. The participants consented to have their interviews recorded.

Prior to interview commencement, the researcher introduced herself and the reasons for doing this study. The participants then were invited to express their feelings, thoughts, and insights relevant to their experiences with IVF treatment, especially their perceptions of the treatment process and the support they offered or received from each other. Interview guide was drafted by the authors. It has then been carefully revised based on the critical comments from qualitative scholars and the results of pilot test. The final version of interview guide contained the following open-ended questions: (1) Would you like to briefly describe the fertility treatments you had undergone? (2) Please describe your reactions and the process of decision-making when it was suggested that you undergo IVF treatment. (3) How did your wife/husband cooperate and become involved in the treatment? (4) Please describe your sharing, communication, and support for each other during the treatment. (5) How did this interaction influence your psychological well-being and your relationship with your partner? (6) What do you think about the support that you received from health care providers, parents, friends, and relatives in the course of your IVF treatment? (7) When an undesirable outcome was uncovered, how did you cope and adjust together (for couples with an unsuccessful cycle)? The field notes have been made during and after the interview.

### Ethical considerations

Ethical approval was obtained from the Human Subjects Ethics Sub-Committee of The Hong Kong Polytechnic University, and permission to conduct the study was sought from the Affiliated Women’s Hospital of Zhejiang University School of Medicine. An explanation was given to the participants of the purpose of the study and its voluntary. The written consent of the participants was obtained before the start of the interviews. Pseudonyms and an indication of their gender, e.g. Fan-F, Xuan-F, Chun-M, were used to protect the participants from being identified, with “-M” and “-F” referring to males and females, respectively. The audio-tapes and narrative transcriptions were kept in a safe place accessible only by the research team via a password. These materials will be destroyed after the completion of this project.

Interviews would be stopped immediately if the participants experienced psychological distress. The hospital had an experienced psycho-counselor available to help the participants if they reported any psychological discomfort during or after the interviews. None of these couples interviewed in the study had to use the service.

### Data analysis

Data were analyzed adopting the approach of conventional content analysis, to describe a phenomenon [19]. The information collected from the interviews was transcribed verbatim. Two researchers independently read through the data several times to obtain a general impression of the information. Discrepancies between researchers were resolved by discussion. The units of the analysis were selected and coded, then organized into sub-themes and themes. A further data check of the interviews was performed to ensure that data have been thoroughly covered and described, and that the themes accurately represent the topic being studied.

## Results

### Demographic characteristics

A total of 16 couples were approached in this study, but four couples with unsuccessful outcomes refused to be interviewed. They expressed their unwillingness to recall their psychological trauma. As a result, a total of 12 couples, 3 with failed cycles and 9 with successful outcomes were recruited. Three of 9 couples had previously experienced failed cycles, and 6 couples were successful in their first treatment. The mean age of the female participants was 32.2 years (range, 28–40 years old), while that for the male was 35.6 years (range, 30–44 years old). The participating couples had been married for an average of 5.95 years (range, 3–14 years). Two couples believed in Buddhism, while the others had no religion. The education levels of the men and women were the same, with 33.3% having received a bachelor’s degree, 41.7% a junior college degree, and 25% a high school diploma. The cause of infertility among these couples can be attributed to female factors (66.7%), male factors (16.7%), and a combination of factors (16.7%). The couples had received infertility treatments for an average of 4.14 years (range, 2–11 years). The average number of IVF cycles tried was 1.82 cycles (range, 1–5 cycles).

**Fig 1:**
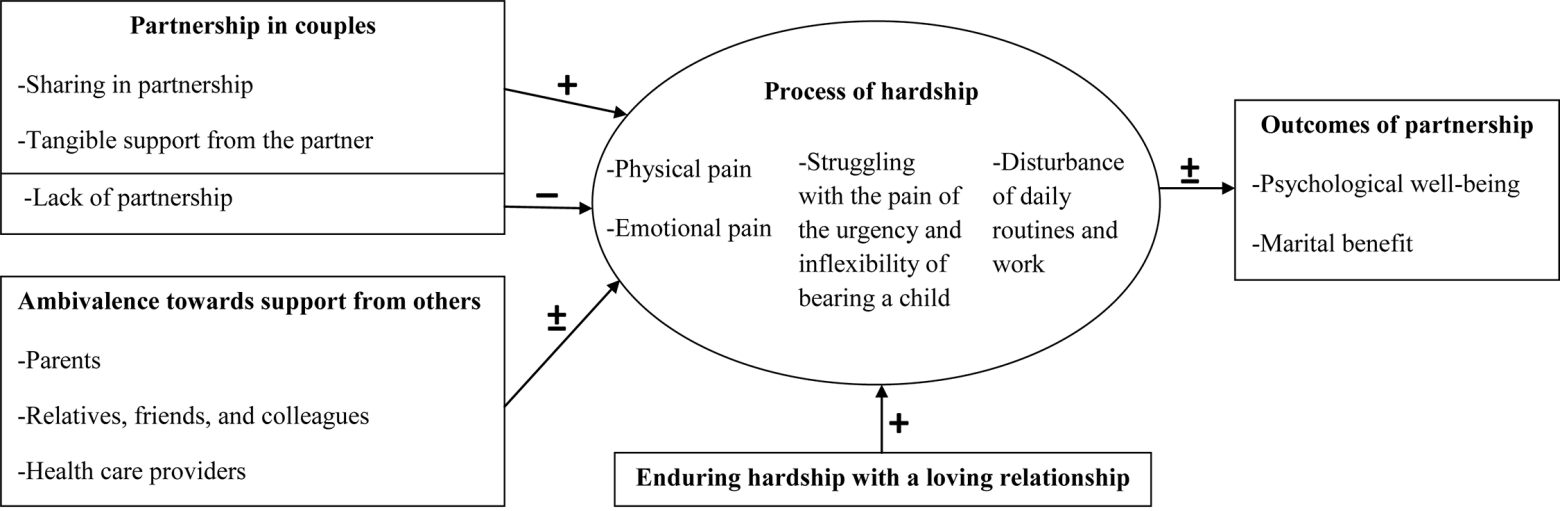
A preliminary conceptualization of the overall experiences of infertile couples undergoing IVF treatment.

The data from the interviews can be categorized into four themes. The major themes and sub-themes emerging from the analysis of the transcripts included: the process of hardship (physical pain, emotional pain, struggles with the urgency and inflexibility of bearing a child, and the disturbance of daily routines and work); enduring hardship with a loving relationship, the partnership of the couples (sharing, tangible support, psychological well-being, and an improved marital relationship as outcomes of partnership, lack of partnership); and ambivalence towards social support. A preliminary conceptualization of the overall experiences of infertile couples undergoing IVF treatment was proposed (Fig 1).

### Process of hardship

This theme describes the infertile couples’ perception of IVF treatment. After entering the torturous and intrusive treatment cycle, the participants underwent a journey of physical and emotional pain during the treatment process. During period when they awaited the results of the treatment, they experienced fear that the outcome might be unfavorable. These treatments also exacerbated the couples’ internal struggles over the issue of bearing a child, and disturbed their daily routines.

#### Physical pain

The majority of women reported experiencing physical pain in the course of the IVF treatment, including from daily injections, intrusive procedures, and the side-effects of medications. The required injections for conventional treatment protocols include the gonadotropin-releasing hormone (GnRH) agonist to prevent premature ovulation, a follicle-stimulating hormone (FSH) to stimulate the development of follicles, human chorionic gonadotrophin (HCG) to facilitate the final maturation of oocytes, and progesterone to support changes in the endometrium. In order to monitor the ovarian response, frequent blood tests and trans-vaginal ultrasound scans were necessary.

One of the women expressed her feelings about the process:

*“The progesterone shot is the worst as it needs to go deeply into muscle and it is oil-based*. *On top of the numerous injections*, *there were also blood tests that made me feel like a pin cushion*. *I would not have wanted this to happen*, *but I have to bear it as I want to have my own baby*.*” (Ke-F)*

One of the husbands could actually “share” his wife’s physical pain, stating that:

*“The injections of progesterone have already gone on for 72 days after the embryo transfer*, *and will continue for at least 20 more days*. *There are more than 400 pinholes in her body*. *If my wife were skinny*, *her buttocks would be badly damaged*.*” (Bin-M)*

Some women described the oocyte retrieval as the toughest procedure, as it is accompanied by an unexpectedly lengthy period of recovery from cramping, bloating, and fatigue.

*“After egg retrieval I had severe vomiting and could not eat anything*. *The enlarged ovarian was like two water bags hanging*, *making it difficult for me to turn over in bed*.*” (Chun-F)*

*“I couldn’t get out of bed for the whole week because of bloating in my lower abdomen*.*” (Qin-F)*

The side-effects of the medications reportedly included weight gain, chest pain, and liver damage. The participants also expressed their concern about the long-terms risks associated with stimulating the ovaries.

*“I suffered from severe liver impairment after use of the follicle-stimulating hormone*.*” (Zhang-F)*

*“I am concerned about the possibility of breast cancer or premature ovarian failure*.*” (Fan-F*, *Qin-F)*

To conclude, women reported of physical pain due to the frequent injections, invasive procedures, and various medication-induced side effects while undergoing IVF treatment

#### Emotional pain

All the women experienced emotional pain and suffered from psychological torture, causing sleep disturbances, frustration, disappointment, and anxiety. A woman actually described the process of IVF treatment as climbing a mountain step by step, with each step being torturous and accompanied by a fear that felt like she was facing an impending death sentence.

*“It was really like climbing a mountain; each step was a psychological torture*, *worse than the physical pain I suffered*. *To me*, *waiting for the results of the pregnancy test was like death sentence*.*” (Chun-F)*

Some women reported sleep disturbances with constant worries about losing the embryo that had been transferred.

*“I could not fall asleep at night*, *worrying that I would not get pregnant*.*” (Jun-F)*

*“I just stayed in bed except to go to the washroom*. *One of the women in the ward even took small steps like an ant*, *for fear of losing the embryo*.*” (Guo-F)*

*“I did not dare to move around*, *but stayed in bed worrying about aborting the embryo*.*” (Fan-F)*

*“From the third day after embryo transfer*, *I became extremely worried because of the repeated negative results of the pregnancy tests that I took by myself*, *and was having nightmares*.*” (Ke-F)*

The disclosure of a negative pregnancy outcome was also described as the most frustrating and painful disappointment. Some couples refused to recall the failed experience and avoided talking about it. A woman had tears in her eyes when she said:

*“I was extremely upset and heart-broken when I had the fifth failed IVF*. *I don’t think other people could understand my feelings*. *I am reluctant to talk to other women in the same ward who were successful*. *It makes me even more frustrated and pained*.*” (Ke-F)*

One of the husbands, who shared his wife’s disappointment and pain, said:

*“We were so disappointed about the unsuccessful result after spending so much energy and effort*. *I can feel the pain that she went through*. *We started with some hope and ended up with nothing*. *It was frustrating and we were hurt*.*” (Hong-M)*

#### Struggling with the pain of the urgency and inflexibility of bearing a child

Even as they suffered from physical and emotional pain, the couples expressed the internal struggles that they went through in wanting to have their own biological child. They were mostly inflexible about their state of childless, and would not accept adoption as an alternative. These infertile couples underwent the hardships of treatment because of their internal struggles over bearing a child and their urgency to have a baby.

Women described their urgency to conceive a child because of their age.

*“I have already failed five cycles*, *and I am now 35 years old*. *There will be a decreased chance for success as the quality of my eggs is getting worse*. *I really don’t have much time*, *so I cannot wait but must go through the treatment*.*” (Ke-F)*

Infertile couples expressed their attitude towards and refusal to accept their childlessness, and insisted on having their own biological child.

*“My life will not be complete unless I can have my own child*. *I will strive and endure whatever physical pain and emotional turmoil I need to go through*, *and pay whatever price I have to pay*.*” (Fan-F)*

Many couples saw no other alternative but to tolerate the physical and emotional pain of the IVF treatment, and were not willing to accept a childless life or to adopt a child.

*“To me*, *adoption is like raising a child for others*. *I would rather go through the pain to have my own*.*” (Guo-F)*

*“There is absolutely no alternative*. *I definitely will not accept adoption as there are too many problems and issues with adopted children*. *I would rather undergo all the pain to have my own or none*.*” (Bin-M)*

In short, couples struggled with inflexibility of childbearing and were unlikely to accept childfree life or child adoption. There was also age-related urgency contributed to the additional stress for women.

#### Disturbance of daily routines and work

After embarking on In Vitro Fertilization treatment, the women had to adjust their daily life, work, and activities because of frequent treatment appointments, medications, injections, and monitoring, causing disruptions to their daily routines.

Those who were from towns or rural areas far from the reproductive medical center had to stay in a hotel during the entire cycle, leading to a relatively isolated life.

*“My hometown is 300 miles away*. *So*, *I had to stay in a hotel once I entered the cycle*. *There was nothing I could do but stay there to wait for the results*. *There was no way that I could return to my daily life with this treatment*.*” (Li-F)*

A woman described how treatment affected her daily routine.

*“My daily normal routines were thrown out of the window*, *especially in the days after the embryo was transferred*. *I could not*, *or more accurately*, *did not even dare to cook a meal for myself*. *Each time I entered the cycle*, *my parents came over to my house to do things for us*. *They took care of me and did all of the housework*, *as my husband has a highly demanding job*.*” (Ke-F)*

The participants stated that the IVF treatment was disruptive to their work. Six women quit their jobs or were reduced to working part time. Other women kept their jobs but frequently took leave for medical appointments. Some described the impact of infertility and the associated treatment on their careers.

*“I could only find a part-time job during the four-year period*. *The process of treatment is too complicated*. *The hospital has almost become my home as I visit it so frequently*. *Each time*, *I have to spend nearly a whole day there*, *waiting for the doctor’s inquiry*, *the test*, *and the results*.*” (Jun-F)*

A husband also commented on the situation that his wife was in before quitting her job.

*“My wife felt guilty and pressured for taking leave so often*. *It definitely influenced the day-to-day running of the company where she worked*. *So my wife ended up quitting her job last year*.*” (Hong-M)*

Over the course of the IVF treatment, the couples experienced hardship. They considered the process to be an arduous trip marked by physical and emotional pain. The women regarded the oocyte retrieval as the most horrible procedure, worse than the injections, tests, and embryo transfer. The waiting period for the outcome of treatment and a negative pregnancy result were the most stressful events–like a death sentence for the couples. Couples also had to alter their daily life, or even quit their jobs, because of the complex and time-consuming treatment. The couples tolerated this hardship because of their internal struggle over the urgency and inflexibility of bearing a child.

### Enduring hardship with a loving relationship

Couples reported that it was their loving relationship that enabled them to endure the hardship. The participants also described the interpersonal skills that they used in their relationship, which helped them to maintain their marriage.

*“My husband and I were best friends for two years before we got married*. *He had confided his fertility problem to me before we got married*. *I would certainly regard it as a couple’s shared responsibility that we should face together*.*” (Gang-F)*

Women said that they would express their feelings directly to reduce misunderstandings with their partner.

*“Sometimes when I am uncomfortable or depressed*, *I will tell my husband my feelings and needs*. *He will respond to me and offer suggestions or merely provide me with emotional support that makes me feel better*.*”* (Jun-F)

Women reported that they would avoid or dissolve conflicts through mutual understanding and a gentle way of speaking.

*“If I could do things by myself*, *I would try not to bother him about accompanying me to the clinic*. *On the other hand*, *my husband has always said that he would like to go through every tough time with me*. *The possible conflict has been minimized as we are considerate of each other*, *which also in turn improves our intimacy*.*” (Zhang-F)*

*“Sometimes*, *when I was not satisfied with my husband’s response to my needs or requests*, *I would not get angry but gently tell him about my disappointment and how it hurt*. *It works quite well with our tender loving relationship*.*” (Chun-F)*

Couples reported that they had a loving relationship before the treatment, which played an important role in their ability to endure the hardships of the treatment. They described using interpersonal skills such as explicitly expressing their feelings, achieving mutual understanding, and cultivating a tender loving relationship.

### Partnership in couples

The infertile couples perceived that they had a “partnership” in facing the hardships of the IVF treatment. Women reported that the sharing of feelings and the giving of tangible support between the two partners was essential for their psychological well-being and the couples’ marital relationship.

#### Sharing in partnership

Couples described their mutual decision to undergo the hardship of IVF treatment and their sharing of the responsibilities. A woman indicted her willingness to undergo treatment with mutual support in their marriage.

*“It was not the decision of one person*. *It was our mutual agreement to undergo the treatment*. *After the torturous egg retrieval procedure*, *we together decided on the maximum number of cycles that we would be willing to try*.*” (Chun-F)*

The husbands indicted the role they played in supporting and sharing the decisions of their wives.

*“As I suffered from azoospermia*, *I initially hesitated to start IVF by donor semen*. *But my wife insisted on having her biological child for us*. *After a discussion*, *we come to a mutual decision on the IVF*.*” (Suan-M)*

*“After so many failed treatments*, *it was suggested that we adopt a child*. *We sat down and discussed our thoughts on adoption*, *and reached the consensus that we would continue treatment instead*.*” (Bin-M)*

*“Unlike my wife*, *I am quite flexible on the issue of bearing a child*. *But I understand her wishes and would support whatever makes her feel better*. *So we will continue the treatment even after five failed cycles*.*” (Shan-M)*

Couples also shared their sorrow and joy with each other about the treatment outcomes.

*“The third day after the embryo transfer*, *the blood test result was not favorable*. *My wife was so depressed and called me immediately*. *I comforted her*, *saying that it does not matter and that we may still have a chance*. *I then picked her up from the hospital*. *It turned out that this cycle was successful*. *We were so happy and celebrated together*.*” (Bin-M)*

*“We have a shared responsibility to endure the painful aspects of life together since we married*. *We shared our sorrow over the failed treatment results*, *but together kept the secret from my parents*.*” (Cheng-M)*

#### Tangible support from the partner

Women reported that they had received tangible support from their husbands, in that their husbands accompanied them to the clinic, assumed some of the housework, and took care of them at home.

*“My husband did almost all of the housework*, *particularly during the two weeks after the embryo was transferred*. *He told me that since I suffered so much in the treatment he could only do some trivial work to relieve my stress*.*” (Jun-F)*

*“Last time*, *I suffered from ovarian hyper stimulation syndrome after being injected with a follicle-stimulating hormone*. *I was frightened*. *Fortunately*, *my husband stayed with me*. *We talked and comforted each other*. *It would have been hard to go through these sufferings without his unfailing support*.*” (Li-F)*

*“I was very well taken care of by my husband during the treatment*. *I was surprised by his caring attitude*. *He even washed my hair when I was confined to bed due to cervical cerclage following our successful cycle*.*” (Wei-F)*

A husband declared that this was the least that he could do for his wife, who suffered so much in their battle to have a baby.

*“As much as possible*, *I escort my wife to the clinic*, *where she undergoes the torturous treatment*. *As we are striving together to achieve a common goal*, *I cannot leave her to fight the battle alone*.*” (Yang-M)*

#### Psychological well-being and marital benefit as Outcomes of Partnership

In going through the hardship of IVF treatment, women reported enjoying psychological well-being and an improved marital relationship because of their partnership in sharing their feelings and giving support. Women described the partnership with their husband as being related to their psychological well-being.

*“My husband has a good attitude*. *When I felt stressed*, *he always directed me to think in a positive way*. *He told me that he was concerned about my physical pain and feelings more than about the outcome of the treatment*. *He cares about my feelings and emotions*, *which gives me much relief*.*” (Li-F)*

*“Whenever I think of his unconditional love and support*, *I feel strong enough to face the difficulties of the treatment and not be so afraid of the failure of the treatment*.*” (Jun-F)*

Through this hardship, some couples reported that their marital relationship had improved.

*“Honestly*, *in the beginning I even complained about his infertility*. *However*, *after we went through various hardships together*, *our love has grown stronger*. *The intimacy and satisfaction in our marital relationship has increased*.*” (Chun-F)*

*“During the process of treatment*, *we supported each other more than we ever had before*, *which led to personal growth and also enhanced our marital satisfaction*.*” (Jim- M)*

#### Lack of partnership

However, not all the couples were lucky to have the involvement and support of their partners. Some women complained of a lack of involvement or partnership on the part of the male partners, even in the presence of their husbands.

*“I think he just did not understand the whole process*. *I made all of the decisions for the treatment*. *He has not offered any help or suggestions*, *but just did what I told him*.*” (Qin-F)*

*“This treatment process was a psychological trauma that both of us were unwilling to discuss*. *(Ke-F)*

Some men complained of a lack of partnership or emotional support from their wives:

*“My wife complained about spending a lot of money because of my fertility problems*. *She was so angry and kept blaming me when the pregnancy result was negative*. *She even said that she wanted to divorce me*. *Although she was not serious*, *I still felt hurt*.*” (Suan-M)*

*“I was quite disappointed when the first cycle was unsuccessful*. *My parents and I stayed with my wife for the whole day and comforted her*. *However*, *I in fact felt equally depressed*, *and I hoped that they realized my pain as well*. *But no one seemed to see that I also needed the emotional support*.*” (Hong-M)*

The infertile couples reported their perceptions of their partnership during the IVF treatment. Couples described their shared decision, responsibility, sorrow, and joy with each other. Women received tangible support from their husbands. It was reported that partnership interactions between two partners can contribute to the psychological well-being of each partner and improve their marital relationship. However, negative partnerships were revealed in the interviews, including a lack of involvement and partnership on the part of the male partners, and a lack of emotional support for males from their wives.

### Ambivalence towards social support

Infertile couples reported that they had received support from parents, relatives, friends, colleagues, and health care providers. They were ambivalent about such support. Some reported that they felt guilty about receiving support from their parents because it seemed to them that they had added to their parents’ burden.

*“As my husband is busy with his job*, *my parents helped to do our housework and took care of me during the treatment*. *Having their support and sharing my feelings with them did a lot to reduce my anxiety*.*” (Guo-F)*

*“My mom treated me like a queen during the treatment*, *which became a source of pressure on me as I added to her burdens*. *Sometimes*, *I wished that I had kept it a secret from her and just kept it between my husband and me so as not to cause my mom to worry*.*” (Wei-F)*

*“In the beginning of my fertility treatment*, *my mother accompanied me to the treatments*. *But I later found that my mom was stressed and worried too much*, *so I decided not to trouble her any more*. *Instead*, *my husband and I*, *as a dyad*, *are supposed to face the hardship together*.*” (Chun-F)*

Some participants intentionally kept their condition and treatment a secret from their friends or relatives, not wanting to hear useless or unintentionally unfavorable comments that would create stress for them. Some even regretted having told others of their treatment and outcomes.

*“My wife’s manager was very supportive*. *She allowed my wife to take leave and told her not to worry*. *After all*, *we could not afford to lose our jobs*. *We were also grateful for the manager’s recommendations on doctors and folk medicine*. *But the support also gave us pressure*, *especially when she constantly asked about the progress of the treatment*.*” (Bin-M)*

*“The less people know about the treatment the better*. *We do not want our child to be treated as abnormal*, *just in case our kid turns out to be abnormal*.*” (Fei-M)*

*“I received a lot of ‘red packets (lucky money)’ from friends and relatives after I was told that I am pregnant*. *But I am still worrying about whether the baby will come to term*. *My uncle then told me that babies conceived via IVF have a higher rate of birth defects*. *I regretted having told him that the baby had been conceived by IVF*.*” (Chun-F)*

Women do generally regard the medical information received from health professionals as beneficial. As one woman stated,

*“We benefit a lot from the health education from the ART center and the booklets on IVF treatment*.*” (Jun-F)*

However, more women complained that during the treatment there was a limited amount of time for inquiries, a lack of involvement in decision making, and a lack of psychological support from health professionals.

*“I have to say that the doctors in the clinic are too busy to answer our inquiries*. *Each time I visit the doctor*, *I prepare a list of questions to ask*, *but I do not find time to ask them*, *or they give me quick answers in the belief that I would not be able to fully understand their explanations*.*” (Li-F)*

*“Sometimes*, *we felt like ‘puppets’ doing what were told by the health professionals*. *There was no time for us to ask questions to get involved in the decision*.*” (Bin-M)*

*“I was so nervous when I underwent my first cycle that I think contributed to the failure*. *I might have been successful in the first cycle if I had received professional psychological support at that time*.*” (Guo-F)*

*“The doctors or nurses at the clinic do not provide the psychological counseling that we need*. *We hope that there is a psychological counseling clinic that can provide us with the support that we need in the hospital*.*” (Chun-F)*

In general, couples were ambivalent about receiving social support, which might either help or lead to further stress. Couples felt guilty due to the extra burden that they had put on their parents. Couples also expressed concern about the comments on the treatment and about children from relatives, friends, and colleagues. Most couples considered the professional support to be inadequate, particularly the psychological support.

## Discussion

This study focused on the experience that couples had with IVF treatment and their perception of it. Four themes were uncovered: the process of hardship, enduring hardship with a loving relationship, partnership in couples, and ambivalence towards social support. Based on the results, three aspects were identified for discussion: the couples’ experience with IVF treatment, the couples’ perception of their partnership, and the couples’ attitude towards social support.

### Couples experienced the hardship of IVF treatment

Not surprisingly, the couples considered the process of treatment as a hardship, which concurs with the results from other studies [9, 20, 21]. Although most of the participants had already undergone numerous investigations, laboratory tests, and even surgical procedures before entering the cycle [4], they still suffered a great deal from the injections, oocyte retrieval, and the side-effects of medication.

However, the women regarded all of these as bearable compared with the emotional torture of the treatment. Due to the uncertainty of the outcome of the IVF treatment and their inability to influence the results, the participants could only wait to receive the results, which usually resulted in disappointment. A woman actually equated the period before the results of the pregnancy test were released to waiting for a death sentence. The feelings of anxiety and fear continued until a healthy baby was successfully delivered.

Couples in China, as in other countries, usually resort to trying IVF after they have exhausted their options pursuing other less intrusive forms of treatment. Alternatives such as adoption or living a childfree life, are not widely accepted [22, 23]. Unlike childless women in Nigeria, who have been found to endure verbal or physical abuse from their husbands or from female members of their husbands’ families [24],women in China are often pressured by their own expectations or those of their parents. The traditional value of carrying on the family line has been so deeply embedded that women usually regard childbearing as a family obligation [7, 23]. All of the women reported suffering emotionally during the waiting period. When the treatment was unsuccessful, this not only led to immediate heartbreak and shock but also to long-lasting psychological trauma. Women who became pregnant still reported suffering from extreme anxiety due to previous failed attempts at IVF. The finding was supported by a study conducted in Sweden [25].

Although there are no age restrictions on women seeking IVF treatments in China, the age-related decline in the quality of the oocytes greatly reduces the success rate of the treatment [26]. Difficulties are still encountered with the sharing of eggs and the practice is not common in China [27]. It was understandable that older women would feel the urgency to bear a child. About one third of couples in Western countries would terminate their IVF treatment after one or two failed IVF cycles [28]. Some Chinese couples in this study had gone through five cycles, and were still determined to go through another cycle.

Many participants in this study were clearly very inflexible about the issue of bearing their own child struggled. Given the importance of childbearing in traditional Chinese culture [7, 23], couples in China insist on having their biological child who can carry on the family bloodline, and refuse to consider adopting a child or settling for a childfree life. The low level of acceptance of adoption by Chinese infertile couples was also supported by a study conducted in Hong Kong [29].

The disturbance to their daily life and work was another burden on couples undergoing IVF treatment. Some women even quit their jobs to pursue the treatments because some working for private companies were unable to take long leaves of absence to make frequent and time-consuming visits to the clinic and to undergo procedures. Similar findings have been reported in earlier studies [9, 30].

### Couples’ perception of partnership when undergoing treatment

It has been reported that the kind of partnership that a couple has can either act as a buffer against or contribute to the hardship of the fertility treatment. The characteristics of partnership that emerged from the data were consistent with those of an analysis of the concept of “partnership” among infertile couples based on a review of the literature [31]. It was reported that “partnership” is a process of joint hardship in that the infertile couples endure the hardship of infertility by sharing their thoughts and feelings and supporting each other. With a partnership, infertile couples would expect to see an improvement in their psychological well-being and marital relationship.

A lack of partnership was observed among some infertile couples. There were complaints of a lack of involvement from male partners. Some women in the present study initiated the fertility treatment and made the decisions themselves, without the involvement of their male partners. The tendency of infertile women to orchestrate the treatment plan was reported in an earlier study [32]. However, this is different from the “uncooperative husbands” reported in Japan, who perceived the co-treatment as a burden [33]. In the Japanese study, some women even deliberately reduced the involvement of their male partners for fear that they would feel pressured. Although in a modern society women have other ways to realize their value, Chinese women still regard motherhood as a major role and a respected identity [29]. Infertile women have a stronger desire to bear a child than men and are more likely to take responsibility for a couple’s infertility [34]. However, if the process of seeking treatment is dominated by the female, this could potentially harm the couples’ partnership, and consequently influence their mental well-being and marital relationship.

If not expressed clearly, a partner’s ourbursts of anger or sadess may be interpreted as blame by the other partner. Infertility serves to magnify the pain that couples experience during their normal life [6]. It has been found that unsatisfactory support between spouses is associated with infertility-related stress [32, 35] and conflicts between partners [36, 37]. Therefore, it is important that infertile couples learn how to communiate more effectively among themselves and be equipped with conflict-resolution skills.

The men in this study also complained that they lacked emotional support, even as most focused on extending suport to their wives. The lack of support for male partners was also reported in another study[25]. It has been widely accepted that infertility affects the couple as a dyad and that among infertile couples the man usually takes up the role of supportive partner during the treatment. Men are usually expected to be strong and suppress their emotions when encountering adversity, which might contribute to depression [38]. Studies have shown that among IVF couples the men also reported symptoms of depression and their needs were mostly neglected [39–41]. It is concluded that among infertile couples the men need to receive support from their partners and from health professionals.

### Couples’ attitude towards social support

This study found that, although the parents of infertile couples could offer some help, the couples preferred to keep the hardship to themselves for fear of adding to their parents’ burden. The couples in this study reported that the support that they received from friends and relatives was minimal, but could be stressful. A study also indicated that support from friends was not correlated with infertility stress for both men and women [42]. Some couples intentionally concealed from others the fact that they had resorted to IVF treatment, to prevent their children from being labeled. Therefore, support from parents, friends, and relatives plays a limited role in the couples’ efforts to cope with the hardships of IVF treatment.

Support from health care providers was described as inadequate because of the short consultation time in the clinic and the lack of psychological support. As another study revealed, the hectic schedule of a clinic makes it difficult for the clinic to satisfy the information and psychological needs of outpatients [9]. This kind of clinic mainly provides information on the medical aspects of infertility and IVF treatment. Counseling or psychosocial interventions are usually not available, and couples have to cope with the difficulties themselves. Research has indicated that support from health professionals is particularly effective at improving the stress and anxiety suffered by individuals who are grappling with the issue of infertility [43]. The results of this study suggested that psychosocial support from health care providers is needed and should be provided to couples together, as dyads undergoing IVF treatment.

### Limitations

There are some limitations in this study. First, nine of the 12 couples recruited achieved a successful pregnancy during the interviews. Although three of those nine couples were asked to share their experience of failed cycles, the uneven distribution between couples who were ultimately successful and those who were not might have introduced a bias to the interview data. Second, one may also concern about the relatively small sample size. Although data saturation was reached, future research with larger sample size is needed so that other factors that might influence infertile couples’ experience can be addressed, and stronger conclusion can be reached. Another limitation is that the couple-based interviews were conducted with the presence of both partners, which might have hindered the free expression of deep feelings. Nevertheless, it was decided to take this approach for the interviews in order to observe the dyadic interaction. The male partners in these interviews usually took a supporting role, in that they were less likely than the female partners to express themselves in the interviews or to confide their sufferings. Future studies should adopt a mixed interview format to explore each partner’s feelings and experiences.

### Implications for mandated psychological counseling or a supportive program

Despite the limitations of this study, it has some implications for health professionals who serve infertile couples and for policy makers. The couples in the interviews expressed a need for psychological counseling; thus, a supportive intervention should be made available to infertile couples as part of the infertility treatment. In Austria, there has been a law since 1992 stipulating that psychological counseling is to be provided to infertile couples [44]. Austrian fertility specialists are obliged to offer psychological counseling or psychotherapeutic care to couples unless the couples refuse such services.

### Implications for the focus, timing, format, and outcome measures of a supportive program

The themes identified in this study will help health professionals or researchers develop a supportive program for couples undergoing IVF treatment in China. The timing of the supportive intervention is important. The findings of this study suggest that psychological support should be given during the time that couples are waiting for the outcome of the IVF treatment, and also when a negative pregnancy result has been disclosed. Other than providing psychological counseling, the intervention should focus on enhancing the couples’ partnership with regard to sharing, support, and communication, and on strategies for increasing the couples’ psychological flexibility and acceptance of the uncertainties surrounding the bearing of a child. The intervention should target infertile couples as dyads, including both the male and the female partner in an infertile couple. To be effective, the intervention should include such outcome measures as partnership, psychological flexibility with regard to the issue of bearing a child, psychological well-being, and an improvement in the marital relationship of the couple.

## Conclusion

This study explored the experiences of Chinese couples undergoing IVF treatment, especially their perception of the process and the support between couples. The four themes that were identified were: the process of hardship, the endurance of hardship with a loving relationship, partnership in couples, and ambivalence towards social support. The findings offer insights into the sufferings of IVF couples and point to the need for a supportive program for infertile couples as dyads to help them get through the hardships of treatment. The findings also provide empirical contributions on the right timing, focus, format, and outcome measures of a tailored program. It is also suggested that policy makers mandate the incorporation of psychological counseling or intervention into infertility treatment. It is hoped that supportive interventions will enhance the partnership of infertile couples undergoing IVF treatment and their flexibility on the issue of bearing a child, resulting in improvements to their psychological well-being and marital relationship.
